# Can People Sleep Too Much? Effects of Extended Sleep Opportunity on Sleep Duration and Timing

**DOI:** 10.3389/fphys.2021.792942

**Published:** 2021-12-22

**Authors:** Elizabeth B. Klerman, Giuseppe Barbato, Charles A. Czeisler, Thomas A. Wehr

**Affiliations:** ^1^Division of Sleep and Circadian Disorders, Brigham and Women’s Hospital and Harvard Medical School, Boston, MA, United States; ^2^Department of Neurology, Massachusetts General Hospital and Harvard Medical School, Boston, MA, United States; ^3^Department of Psychology, University degli Studi della Campania Luigi Vanvitelli, Campania, Italy; ^4^Intramural Research Program, NIMH, Bethesda, MD, United States

**Keywords:** sleep, homeostasis, recovery sleep, sleep restriction, human, insomnia, sleep variability

## Abstract

Many people are concerned about whether they are getting “enough” sleep, and if they can “sleep too much.” These concerns can be approached scientifically using experiments probing long-term (i.e., multi-night) sleep homeostatic processes, since homeostatic processes move the system toward its physiological setpoint (i.e., between “not enough” and “too much”). We analyzed sleep data from two human studies with sleep opportunities much longer than people usually stay in bed (i.e., conditions in which sleep homeostatic responses could be documented): sleep opportunities were 14–16 h per day for 3–28 days. Across the nights of the extended sleep opportunities, total sleep duration, Rapid Eye Movement (REM) sleep duration and non-REM sleep durations decreased and sleep latency increased. Multiple nights were required to reach approximately steady-state values. These results suggest a multi-day homeostatic sleep process responding to self-selected insufficient sleep duration prior to the study. Once steady state-values were reached, there were large night-to-night variations in total sleep time and other sleep metrics. Our results therefore answer these concerns about sleep amount and are important for understanding the basic physiology of sleep and for two sleep-related topics: (i) the inter-individual and intra-individual variability are relevant to understanding “normal” sleep patterns and for people with insomnia and (ii) the multiple nights of sleep required for recovery from insufficient sleep from self-selected sleep loss is important for public health and other efforts for reducing the adverse effects of sleep loss on multiple areas of physiology.

## Introduction

“Can I sleep too much” is a common question. Epidemiologic data indicate that people who habitually sleep more than 10 h at night are at greater risk of death and other effects [e.g., (Kripke, 2004)]. Because of these correlations between longer sleep duration and adverse effects, many people are concerned that sleeping too much may be dangerous. In one study, once appropriate control for medical conditions (e.g., sleep disorders or severe illness, both of which may increase sleep duration) was conducted, however, the link between long sleep duration and adverse effects was lost (Van Cauter et al., 2008). In addition, no causal links have been established and no controlled laboratories of chronic extension of sleep duration have demonstrated adverse effects. In contrast, controlled laboratory studies have demonstrated causal links between chronic insufficient sleep and multiple adverse effects [e.g., (Scott et al., 2006; Van Cauter et al., 2008; Mullington et al., 2009)]. Yet people still ask sleep researchers and clinicians: can we sleep too much? Is sleep like eating—such that you can eat too much and become overweight? Can one over-sleep? Would over-sleeping have negative health effects? To address these questions, we consider “too much” to be a concept related to physiological homeostasis (i.e., ability to maintain equilibrium).

Sleep timing and homeostasis are regulated within and across multiple days. The physiology underlying this regulation can be studied by shortening or extending sleep and wake opportunities under controlled conditions and monitoring changes in the amount and time course of changes in sleep metrics [e.g., total sleep time (TST), Non-Rapid Eye Movement (NREM) sleep, Slow Wave Sleep (SWS), or Rapid Eye Movement (REM) sleep]. To date, most experiments have focused on shortening sleep and/or extending wake; sleep extension protocols, however, can also provide information about sleep homeostasis (e.g., sleep need, sleep capacity, and sleep satiety).

In healthy individuals without a sleep disorder, the time courses of sleep metrics during both chronic sleep restriction (i.e., multiple days with insufficient sleep) and chronic sleep extension (i.e., multiple days with more time available for sleep than actual sleep duration) would be expected to depend on: (i) prior sleep/wake history, including whether any preceding sleep loss was from acute sleep deprivation (i.e., one extended wake episode) or chronic sleep restriction, since the two have different time-courses of recovery (Cohen et al., 2010); and (ii) the timing, duration, and number of the bedrest episode(s). The time-course and content of sleep during extended sleep opportunities can be used to quantify sleep capacity, which we define as whether someone can sleep when they are not “tired” (e.g., when bored) or as recovery from prior insufficient sleep. These questions are important for documenting basic physiology and for public education about the consequences of sleep loss and how to recover from sleep deficiency, which is formally defined as “deficit in the quantity or quality of sleep obtained vs. the amount needed for optimal health, performance and well-being” by the 2011 NIH Sleep Disorders Research Plan (National Center on Sleep Disorders Research, 2011).

The design of previous protocols has not allowed full documentation of recovery sleep after insufficient sleep for multiple reasons. Firstly, most studies have only one or two nights of recovery sleep, which may not be sufficient. Secondly, many previous studies of extended sleep opportunities may not provide sufficient time in bed (during each bedrest episode) for some individuals to obtain complete recovery from their prior sleep loss, such as scheduling only 10 h time in bed (TIB) (Roehrs et al., 1989, 1996; Harrison and Horne, 1996; Arnal et al., 2015; Skorucak et al., 2018) and/or allowing self-selection of the extended sleep duration (Kamdar et al., 2004), including one study (Webb, 1986) that allowed 12 h TIB for one night, but only 4 of the 42 individuals stayed in bed that long. Thirdly, nocturnal opportunities alone may not provide optimal opportunity for obtaining recovery sleep; more sleep may be obtained if both nighttime and daytime (e.g., nap) opportunities are allowed. Finally, for the night or nights prior to the extended sleep opportunity, either TIB (during which participants try to sleep) or actual TST, have not been reported in most studies; such information is necessary for quantifying the relationship of recovery sleep metrics to prior sleep loss. When the amount of sleep deprivation or restriction prior to the extended sleep opportunity is not known, parameters of homeostatic drive and recovery cannot be calculated. One of our studies, with 16 h of opportunity (12 h at night and 4 h during the day) (Klerman et al., 2004; Klerman and Dijk, 2008) did document each individual’s TIB history for approximately 3 weeks prior to the intervention schedule; those results are examined in detail below. In addition, alertness- or sleep-promoting substance use have not been documented in many studies and so the sleep obtained may differ depending on whether there is withdrawal from those substances. Prior TIB and drug (e.g., caffeine, alcohol) use may be highly variable within and across participants, and may affect the amount of sleep they can obtain during sleep opportunities. Given that individuals may choose to arise from bed for physiological and psychological reasons before their sleep homeostatic drive is fully dissipated, therefore, the design of a study of the effects of extended sleep opportunity should ideally include documentation of sleep history, use of substances that may affect sleep or wake, and enforcement of time in bed during the entire scheduled bedrest episode(s) for each participant.

To address this question of whether it is possible to sleep “too much,” therefore, we reanalyzed data from two relevant studies that included most or all of the conditions cited above. The first study was designed to assess photoperiod, and only hence only scheduled people to sleep at night during an extended episode of darkness each day (Wehr, 1991; Wehr et al., 1993); it included 28 nights of inpatient 14-h scheduled TIB in darkness from 6 pm–8 am. These studies documented increased total sleep time including earlier sleep onset, and increased duration of sleepiness and of melatonin secretion in individuals under conditions of more hours of darkness and sleep opportunity at night. The second study (Klerman et al., 2004; Klerman and Dijk, 2008) protocol both extended the time in bed at night and provided the opportunity for an afternoon nap: it included 1 week at home of scheduled TIB based on the prior 1–2 weeks for each individual and 3–8 inpatient days of a 24-h schedule that included 12 h of scheduled TIB + 4 h wake + 4 h scheduled TIB + 4 h wake with midpoint of the nighttime bedrest episodes the same as the midpoint of the first inpatient bedrest episode that was based on each participant’s at-home schedule. Caffeine, tobacco, and other alertness- or -sleep-promoting substances were not allowed the 2 weeks before or during the inpatient protocol. These studies also documented increased total sleep time during both nocturnal and daytime (nap) sleep episodes compared to an individual’s self-selected sleep times at home; this sleep amount decreased over the days of the protocol.

## Materials and Methods

### Participants

Study 1: 15 adults (20–36 years; 1F:14M) participated. Healthy volunteers were screened with interviews, physical examinations and routine laboratory tests and procedures. None had medical or psychiatric illnesses, and none had taken any medication for at least 3 weeks before the studies.

Study 2: 35 adults (18–32 years; 18F:17M) participated. All were healthy by history, physical exam, laboratory tests of blood and urine, a clinical polysomnogram (PSG) for sleep disorders, and a visit with a clinical psychologist. None were using prescription or non-prescription medications for at least 3 weeks before the start of the inpatient protocol and no caffeine, alcohol, or tobacco were allowed 2 weeks before the start of the inpatient protocol; urine toxicology screens were performed during those 2 weeks and at admission.

For Study 1, the protocol was approved by the NIH Intramural Research Program IRB; written informed consent was obtained. For Study 2, the protocol was approved by the Partner’s Healthcare IRB; written informed consent was obtained. Both protocols were conducted before the clinicaltrial.gov requirement.

### Methods

Study 1: Participant sleep/wake schedule and caffeine or other drug consumption before each inpatient segment of the protocol was not regulated or recorded, except that daytime naps were prohibited. The protocol included: (i) 7 nights of inpatient 8-h scheduled TIB in darkness from midnight—8 am, (ii) a constant routine (i.e., enforced wakefulness with multiple small meals under dim light conditions for ∼ 40 h), (iii) 2 weeks of unregulated and unreported sleep/wake schedule at home; (iv) 28 nights of inpatient 14-h scheduled TIB in darkness from 6 pm–8 am and unregulated activities outside the laboratory from 8 am–6 pm; ending with (v) a constant routine. For part (iv), the 6 additional hours of sleep opportunity were added to the beginning of inpatient bedrest episode compared to part (i) (Supplementary Figure 1). This analysis only includes the data from the 28 nights of inpatient 14-h TIB (section iv).

Study 2: Participants documented their self-selected sleep/wake schedule for 2–3 weeks prior to the inpatient stay using a paper-based log, a wrist-worn actigraph, and calls to a time-stamped recording. The last week prior to the inpatient stay and the first inpatient scheduled bedrest episode were at the average onset and offset times of the prior 1–2 weeks for each individual. The first inpatient day had a set of 5 multiple sleep latency tests (MSLTs) (Carskadon, 1986) beginning 2 h after awakening. The next 4 days had a 24-h schedule that included 12 h of scheduled TIB + 4 h wake + 4 h scheduled TIB + 4 h wake with midpoint of the nighttime bedrest episodes the same as the midpoint of the first inpatient bedrest episode. Those 4 days were followed by an intervention/control (no intervention) and 4 more days of 12-h TIB + 4-h wake + 4-h TIB + 4-h wake schedule. The inpatient protocol ended with a 12-h scheduled TIB, a set of 5 MSLTs, and a final 8-h TIB (Supplementary Figure 1). Only the inpatient data from days with 16-h of TIB prior to the intervention or with no intervention (control) are included in this report; 3–8 days of data are available for each participant.

### Quantification and Statistical Analysis

Polysomnography records were scored in 30 s epochs using established criteria (Rechtschaffen and Kales, 1968) as non-REM (NREM) sleep stages 1, 2, 3, or 4; Rapid Eye Movement (REM) sleep, Wake, and Other (e.g., Movement Time or Unscorable). Slow Wave Sleep (SWS) was defined as NREM Sleep stages 3 and 4. Each site scored its own recordings. The scored data from both studies were re-analyzed as follows:

Bedrest episodes were defined as the time between lights out and lights on; sleep episodes were defined as the time between sleep onset and sleep offset (Czeisler et al., 1980a), which in this study were calculated using Persistent Sleep (PersistSlp) onset and Final Wake, respectively. PersistSlp onset was defined as the first epoch of 10 consecutive minutes of any stage of sleep after lights out time; Final Wake was defined as the number of consecutive minutes of Wake before lights on; Sleep Episode Duration was defined as the time between PersistSlp onset and Final Wake onset; Wake within the Sleep Episode was the total number of minutes of Wake within the Sleep Episode Duration; Wake after persistent sleep onset (WAPSO) was calculated as the number of minutes of Wake between PersistSlp onset and lights on time; and total sleep time (TST) was defined as the total number of minutes of NREM S1 + S2 + S3 + S4 and REM sleep within the bedrest episode. Metrics were also computed as the difference from the value for that individual on each day of extended sleep opportunity and the first day of extended sleep opportunity. Data from bedrest episodes with > 5% missing data or > 5% stage Other were not included in analyses. For Study 2, data from each 12-h bedrest episode and the following 4-h bedrest episode were combined to create a “daily” value; only data from when both 12 and 4-h bedrest episodes were usable (i.e., not excluded using above criteria) were used for that metric.

For analysis of the time-course within a bedrest episode, data were examined relative to lights out. Data were binned in 30 or 60 min bins and plotted relative to the middle of each bin.

Data were analyzed for all nights of extended sleep for Experiments 1 and 2; for the first 7 and last 7 nights of Study 1; and for the last 14 nights of Study 1.

For all analyses, data were combined within participants and then across participants. 5th, 25th, 50th (median), 75th and 95th percentiles were calculated due to the relatively small sample size and non-Normal/Gaussian distributions of data. Formal comparative statistics were not performed if data medians (50th percentiles) of one protocol were between the 5th and 95th percentile of data from the other protocol. All statistics were performed using SAS for Windows.

## Results

### Outpatient Sleep Durations

For Study 2, average-per-individual TIB durations prior to the inpatient study were 6.1–10.3 h. Outpatient TIB or sleep duration information was not available for Study 1.

### Analysis by Entire Bedrest Episode as Extended Sleep Opportunities Continue

**FIGURE 1:**
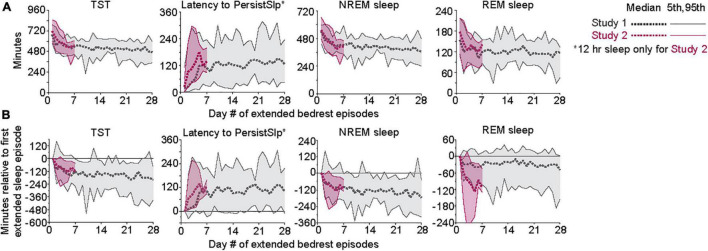
**(A)** Patterns of TST, Latency to PersistSlp, NREM Sleep, and REM Sleep per 24-h across days of both Studies. Gray lines are data from Study 1; red lines are data from Study 2. For each experiment, 5th, 50th (median), 7 and 95th percentiles are shown. Data for NREM Sleep Stage 1, NREM Sleep Stage 2, SWS, Wake in PersistSlp. Final Wake Duration, and Sleep Episode Duration are in Supplementary Figure 2. **(B)** As in panel **(A)** except all values relative to the value for each individual’s first night value.

Patterns of all sleep metrics were similar between the two protocols. Daily (i.e., night episodes only for Study 1; night + day episodes for Study 2) TST, NREM sleep, and REM sleep but not SWS (Figure 1 and Supplementary Figure 2) were markedly higher in the first days of extended sleep opportunity (Table 1). The median values (across all participants) for these three metrics decreased over the first 7 days and then appeared to stabilize. Persistent Sleep (PersistSlp, 10 consecutive minutes of any stage of sleep) latency and Sleep Episode Duration (time between PersistSlp onset and Final Wake) both increased over the first two nights (i.e., night bedrest episodes only for both Study 1 and Study 2) and then appeared to stabilize: after the first ∼7 days, latency to PersistSlp was approximately stable at ∼90–120 min in Study 1 and ∼60 min in Study 2 (Figure 1 and Supplementary Figure 2). The time constant and steady-state values from an exponential fit for TST, NREM sleep, and REM sleep were similar in both studies (Table 2); of note, the fit steady-state value for TST was more than 8.5 h (which would require a TIB of more than 8.5 h). The time courses of other variables were not appropriate for an exponential fit (see Figure 1). The group median of TST, Latency to PersistSlp, NREM Sleep, and REM sleep remained approximately constant after the first ∼7 days through the end of the 28 days in Study 1 (Figure 1).

**TABLE 1:** Difference in minutes between values on night 8 vs. night 1 for Study 1.

Variable	Mean (Std dev) Difference (min.)	*P* value	Minimum, Maximum Difference (min.)
TST	−144.6 (91.4)	<0.0001	−353.5, 10.5
NREM sleep	−113.1 (65.2)	<0.0001	−258.0, 15.0
REM sleep	−31.5 (39.4)	0.008	−114.0, 21.0
SWS	−5.6 (33.3)	0.348	−58.0, 38.5
WAPSO*	50.2 (106.6)	0.090	−134.0, 172.5
LatPersistSlp*	−89.2 (68.4)	0.0002	−18.5, 192.0
Final wake**	39.2 (35.8)	0.0008	0.0, 104.5
SED*	−128.4 (20.5)	<0.0001	−239.0, −12.5

*diff is bimodal, not normally distributed.

**diff is left skewed, not normally symmetrically distributed.

**TABLE 2:** Approximate steady-state values from exponential fit.

Variable	REM sleep	NREM sleep stages 2, 3, and 4	TST
**Study 1**			
Time constant (days)	0.8	3.7	3.1
Steady state value (hours)	2.0	5.9	8.6
**Study 2**			
Time constant (days)	(n.s.)	1.5	1.5
Steady state value (hours)	(n.s.)	5.7	8.9

*All parameters are at p < 0.01 by fit.*

*Exponential fits were not appropriate for other sleep stages.*

**FIGURE 2:**
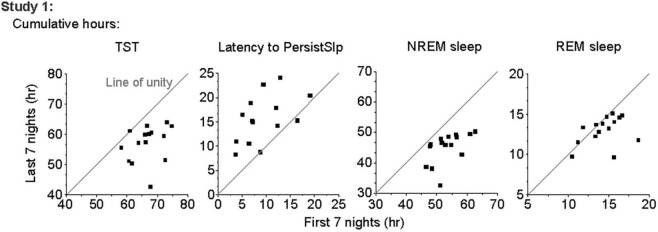
Comparison of total number of hours of TST, Latency to PersistSlp, NREM Sleep, and REM Sleep in first vs. last 7 nights of Study 1 with line of unity (solid line) shown.

In Study 1, all (15/15) participants had higher total duration of TST, NREM sleep, and Sleep Episode Duration and 12 of the 15 participants had higher total duration of REM sleep in the first 7 nights than the last 7 nights of the protocol (Figure 2). Total SWS duration was not higher in the first 7 nights than the last 7 nights. The difference between the first and last 7 nights in TST, Sleep Episode, Duration and REM sleep duration was negatively correlated with the amount in the first 7 nights (i.e., greater amount in first 7 nights was associated with less difference between first and last 7 nights).

### Analysis by Changes Within Each Bedrest Episode

**FIGURE 3:**
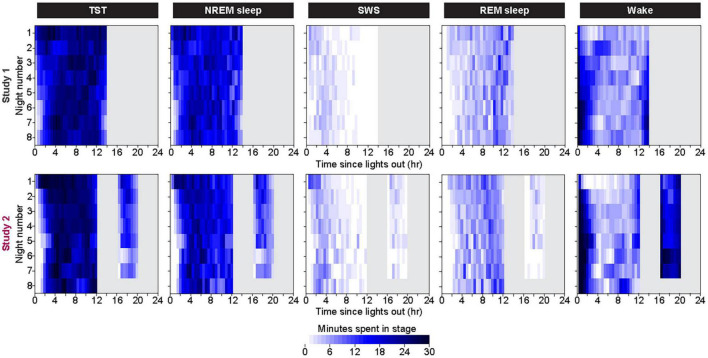
Color heat maps of 30-min bins for the median (across all participants) for TST, NREM Sleep, REM sleep, and Wake for each day of the protocol. Top row contains data from first 8 days of Study 1 and bottom row contains data from all 8 days of Study 2. For ease of viewing, data on each line are plotted as relative to time of lights out for the main bedrest episode. Clock time for Study 1 time of lights out was 1800–0800. Study 2 had bedrest episodes scheduled relative to each individual’s habitual bedtime. Color heat maps for the entire 28 days of Study 1 are in Supplementary Figure 3.

Within each bedrest episode (Figure 3 and Supplementary Figure 3), the highest group median amount of NREM sleep and SWS occurred near the beginning of the bedrest episode and shifted slightly later across the first 7 days, as the amount of Wake at the onset of the bedrest episode increased over the first 3 nights and then appeared to stabilize in both protocols. REM sleep remained highest near the end of the nocturnal bedrest episode across all nights in Study 2. The group median did not show a prominent Wake bout in the middle of the extended night in Study 1, since extended mid-sleep-episode Wake bouts were seen only in some individuals at variable times in some nights (Supplementary Figure 4).

### Analysis by Changes Across Bedrest Episode for the Last 14 Nights of Study 1

**FIGURE 4:**
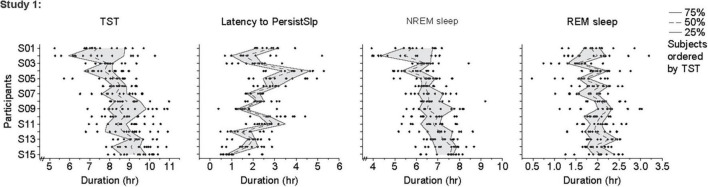
Individual data points, 25th, 50th (median) and 75th values for hours of sleep TST, Latency to PersistSlp, NREM Sleep, REM Sleep in the last 14 nights of Study 1. Data for Latency to PersistSlp, NREM Sleep, REM Sleep are organized by TST duration.

For the last 14 nights of Study 1 (Figure 4 and Supplementary Figure 5), there was large night-to-night variability in multiple metrics: there was a range of ∼20–100% in single night values relative to median values in TST (median range 185.0 min, median of medians 518.3 min, median of ratio [=(max-min)/median] across participants for 14 nights of 0.4), Latency to PersistSlp (range 112.5 min, median value 112.5 min, average ratio 1.0), NREM sleep (range 157.0, Median 392.5, Ratio 0.4) SWS (range 28.5 min, median 35.8 min, ratio 0.9), REM sleep (range 71.0 min, median 119.5 min, ratio 0.6), WAPSO (range 178.5 min, median 162.3 min, ratio 1.0) (Figure 4). The variation between individuals seen in median TST appears to be mostly due to due to changes in median NREM sleep more than median REM sleep. There were relationships between median value in the last 14 nights and variability, with negative correlations between median and range (75–25%) for TST (correlation = −0.54), NREM Sleep (correlation = −0.41) and WAPSO (correlation = −0.41), a positive correlation for REM sleep (correlation = 0.29), but not LatPersistSlp (correlation = 0.01).

**FIGURE 5:**
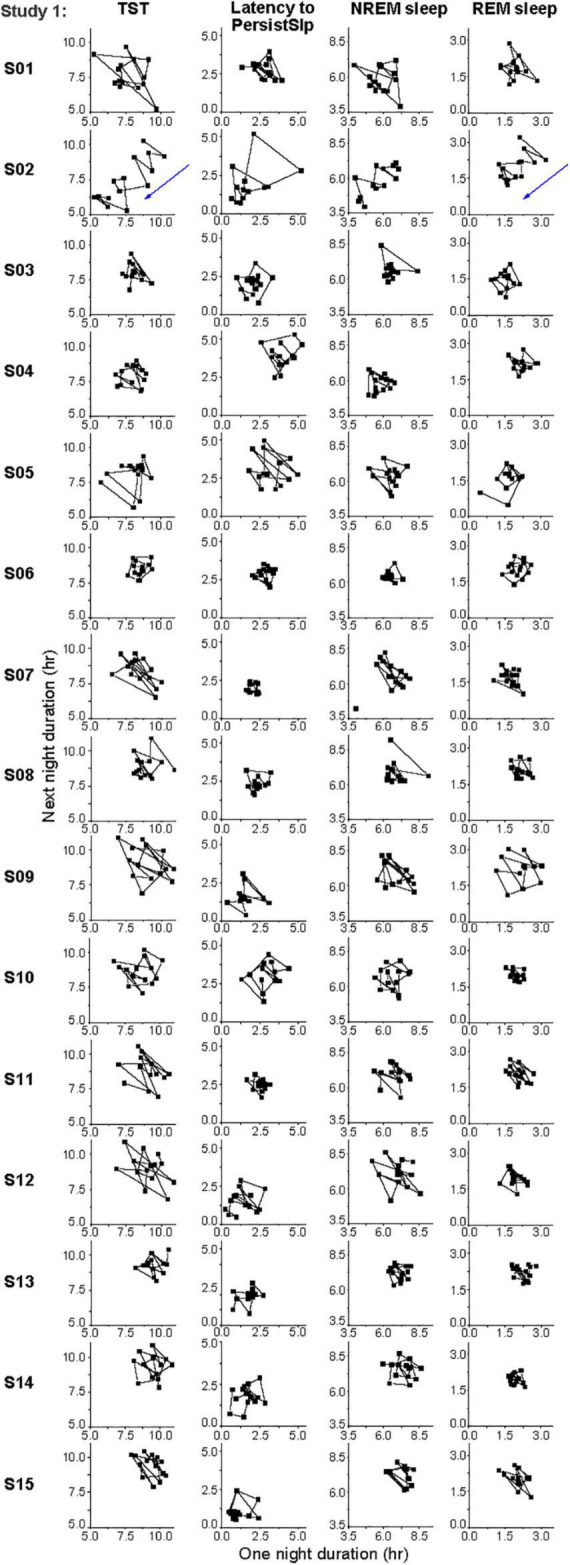
Individual daily variations in hours of TST, Latency to PersistSlp, NREM Sleep, REM Sleep in the last 14 nights of Study 1. Consecutive current day value vs. next day value (both in hours) are plotted for each individual. Participant numbers are the same as in Figure 4.

There was no obvious consistent 2-night or 3-night periodicity in this variability, suggesting that short-term (e.g., one or two night) sleep homeostasis was not a major factor (Figure 5). However, analysis of the night-to-night differences demonstrates homeostatic regulation is present: there is a negative correlation for the night-to-night change in duration of TST, NREM sleep, and REM sleep or of Latency to PersistSlp: a larger difference between consecutive nights is followed by a smaller difference and vice-versa (Supplementary Figure 5).

## Discussion

In response to the question: “Can I sleep too much?,” the answer is “No,” since “too much” implies sleeping longer than is biologically necessary. We demonstrate that the average healthy young human can not chronically sleep over 10 h per day: the average sleep duration was 8.6 h over the last 14 nights of the Study 1 protocol. Individual night TST, however, ranged from 5.2–11.0 h. Two other studies support our observed homeostatic set-point value of 8.6 h: in one study in which healthy individuals had scheduled 8-h bedrest episodes (Van Dongen et al., 2003), there was a progressive decrease in visual vigilant performance (suggesting insufficient sleep), while participants with 9-h bedrest episodes in another study (Belenky et al., 2003) did not have this decrease.

In this report, we compared data from two conditions with extended sleep opportunity: one with one 14-h nighttime bedrest episode and one with 16 h of sleep opportunity split into 12 h at night plus 4 h during the day. For both studies, the values of sleep metrics in the first few days suggested rebound recovery (i.e., a homeostatic process) from sleep loss: when scheduled to an extended sleep opportunity, there were initially increased values of TST, NREM sleep, and Sleep Episode Duration and decreased sleep latency that lasted 1–3 days. This was followed by decreasing values of TST, NREM sleep, and Sleep Episode Duration and increasing sleep latency for the next few days. This pattern is consistent with a homeostatic process, likely reflecting recovery from prior behaviorally-induced insufficient sleep (or sleep debt). If any extra sleep obtained were voluntary (e.g., due to extra time in bed without any stimulation) and not because of a homeostatic process, then the initial increased total sleep duration would be expected to continue until the protocol ended, and not show the time-course of decaying to a new set point. Indeed, given that after the first few nights, participants were often awake in bed for more than 1 h (total per night), such voluntary sleep might have been preferred. The values and time-courses of recovery were remarkably similar in both protocols. Consistent with our data, another report documented increased TST and REM time during 10-h sleep extension when compared to the 8 h baseline sleep opportunity (Skorucak et al., 2018). Previous reports of “banking” sleep (Rupp et al., 2009), probably reflected recovery from prior sleep restriction. A sleepy feeling after awakening, especially from a nap, may occur in some people, but this does not mean that too much sleep occurred; this feeling is associated with sleep inertia and dissipates as wakening continues [e.g.,(Jewett et al., 1999)].

### Total Sleep Time (i.e., Nighttime Sleep Duration)

There are multiple potential explanations as to why this presumed sleep debt was not repaid in one bedrest episode. (i) The duration of sleep depends on the circadian phase at which the sleep episode is initiated (Czeisler et al., 1980b). The circadian wakeup signal in the morning may therefore end the sleep episode before sleep need is fully dissipated. Note that individuals may be able to sleep past this circadian wakeup signal if they still have a high sleep pressure at that time. (ii) There is a limit to the amount of sleep a person can obtain in a single sleep episode because other homeostatic processes (e.g., hunger, bladder fullness) may interfere with continuing to sleep. (iii) Allostasis (i.e., changing homeostatic level) is present; one mechanism of this would be a change in receptor sensitivity (Phillips et al., 2017). (iv) Previous reports suggested the possibility of core vs. optional sleep (Horne, 1988). This hypothesis is based on studies in which there was sleep restriction followed by *ad lib* sleep. However, in those studies the *ad lib* sleep conditions included self-selected sleep durations in the real world (i.e., not laboratory conditions) and therefore the amount of sleep obtained may have been less than under the conditions of Studies 1 and 2. (v) If sleep restriction squeezed Evening (E) and Morning (M) oscillators (Wehr, 2001) together, as if the individual were living on an extreme photoperiod (as at home before inpatient portion of Study 1), then several days may be required for the two oscillators to expand to another phase relationship, with the associated change in sleep duration. Further work is required to test these explanations.

### Sleep Latency

Study 1 participants had overall shorter Sleep Latency than those in Study 2 for the nighttime bedrest episodes. This may be due to the different prior wake durations in the two experiments (10 vs. 4 h, respectively). Participants in both experiments had increasing Sleep Latency over the first few bedrest episodes. There are multiple possible reasons for these results, including decreased sleep pressure as recovery proceeds, and changes in timing of melatonin onset to a later time, since both protocols include scheduled sleep in darkness ending at a later time (i.e., later in the morning) than the participants had before entering the inpatient portion of the protocol. It is possible this delaying pattern also reveals the inherent onset of the sleep propensity rhythm once sleep debt is partially recovered. When individuals are at home in our industrial world, such a circadian onset might not be seen because of prior sleep debt and because indoor light from TV/room/computers may suppress the circadian signal to go to sleep and also phase delay the circadian system. Conversely, symptoms associated with chronic sleep loss might be synergistic with the circadian signal to sleep, thereby decreasing Sleep Latency when individual are living at home with self-selected sleep schedules.

### Slow Wave Sleep

It is not known why there is almost no SWS in some of the individuals in Study 1. All data in Study 1 were scored by the same individual, though there were different scorers for Study 1 and Study 2. One possibility is that, according to Rechtschaffen and Kales (1968), SWS is defined by a conservative amplitude criterion (0.5–2 Hz, peak to peak amplitude greater than 75 μV), reduced “sleep pressure” during sleep extension might have contributed to a decreased amplitude (below the necessary threshold level) (Skorucak et al., 2018). SWS has also a great inter-individual variability (Tan et al., 2001). Age, gender, BMI and race have also been described as factors affecting SWS (Tucker et al., 2007; Tomfohr et al., 2010); Study 1 did not have a large enough sample size to determine if any of these variables were important. The very low amount of SWS at levels unexpected in a healthy population in many participants in Study 1 makes comparison between the studies difficult.

In Study 2, SWS decreased over the first 7 days of study, suggestive of recovery process, as seen in TST, above.

### Rapid Eye Movement Sleep

There is a striking increase in REM sleep in Study 2 over baseline that then decreases over the first few nights (similar to TST and NREM sleep), suggesting a rebound phenomenon from levels during sleep episodes prior to the study; a similar decline is seen in data from Study 1 (for which baseline data are not available). This raises the question of whether healthy individuals in society are REM-sleep deprived in their habitual sleep: within an 8-h (or shorter) sleep opportunity, there may not be sufficient time for REM sleep or at least the amount received during a longer sleep opportunity. Additionally, since light at night delays circadian rhythms [e.g., (Chang et al., 2015)] and there is strong circadian regulation of REM sleep such that highest levels are expected in early morning (i.e., near the end of a normally timed sleep episode) (Czeisler et al., 1980c), there may be less time for REM sleep to be expressed before the individual awakens after a self-selected 8 (or fewer) hours by alarm and before sleep would otherwise end. A rebound in REM Sleep was also observed after space shuttle missions, during which sleep was ∼6.5 h in duration and had more wakefulness and less SWS in the last third of the bedrest episodes (Dijk et al., 2001).

It is interesting to contemplate the significance of individuals living under conditions of never getting enough TST or REM sleep, which may be a feature of modern life. Acute sleep deprivation (Wehr, 1990; Leibenluft and Wehr, 1992; Giedke and Schwärzler, 2002; Benedetti and Colombo, 2011) and REM sleep deprivation (Vogel, 1980) are both known transient anti-depressants, and most antidepressants suppress REM sleep (Azumi and Shirakawa, 1982; Wilson and Argyropoulos, 2005). On the other hand, in normal participants, chronic sleep restriction (sometimes called partial sleep deprivation, such as we hypothesize occurred prior to the inpatient studies) causes mood deterioration (Motomura et al., 2017a). Participants in Study 1 (Wehr et al., 1993) reported improved mood and energy, and decreased fatigue during the 28 days of 14-h time in bed compared with the 1 week of 8-h time in bed. Motomura et al. (2017b), have recently shown that sleep extension (after self-imposed chronic sleep restriction) improves mood regulation *via* prefrontal suppression of amygdala activity.

Given the longer sleep durations in these studies, there was no evidence of competition (Carskadon and Dement, 1980; Duncan et al., 2009) between REM sleep and NREM sleep during the first days of recovery sleep. In these two protocols, however, there are longer bedrest episodes so both more NREM sleep and REM sleep can occur after sleep loss.

### Patterns Across the Bedrest Episodes

For Study 1, the schedule had extra sleep opportunity with darkness beginning earlier than habitual times, which may have advanced the circadian rhythms and/or onset of melatonin secretion in those participants (Wehr, 1991). For Study 2, sleep was scheduled such that the mid-sleep times were consistent between outpatient and inpatient portions of the study. This may have delayed circadian timing since this schedule delays the time of lights on.

For Study 1, an evening wake maintenance zone (i.e., time when bedrest episodes are not usually initiated) was observed in some people [e.g., 2 people in the 1993 report (Wehr et al., 1993)] and in the overall average. In some individuals on some nights, there was prolonged Wake (plotted as 0 min of TST in Supplementary Figure 4) in the middle of the night, after Persistent Sleep had begun, but the timing of this waking episode was not consistent within and across participants, and therefore is not seen in overall averages. There are historical references of such segmented sleep patterns (Ekirch, 2005). There may have been an evolutionary advantage for a population in which someone is awake at all times. This bimodal pattern also may reflect different movements during the night of dual evening and morning (i.e., E and M) circadian oscillators (Wehr et al., 1995).

There were large inter-individual and intra-individual differences in all sleep metrics (Figures 4, 5 and Supplementary Figure 4). To what extent these differences in Study 1 are due to different behaviors (e.g., caffeine use, light exposure) during the ∼10 h each participant was not in the research facility and/or differences in physiology is not known. Two potential reason for this intra-individual variability are (i) sleep intensity (e.g., as measured by delta power in the EEG) and (ii) the combination of the variability of duration of NREM-REM sleep cycles and the increased probably of awakening from REM sleep (vs. NREM sleep) (Dijk et al., 2001). Reasons for this intra-individual variability should be investigated. Longer-term intra- and inter-individual variability is affected by photoperiod in humans and other animals, with longer sleep episodes across the month (with nighttime light from the moon) (Casiraghi et al., 2021) and during seasons with longer durations of darkness [e.g., (Wehr, 1991; van Hasselt et al., 2020)].

Given that none of these participants had a history of insomnia disorder, it is noteworthy that during this extended sleep opportunity protocol, many of the participants exhibited sleep patterns similar to those of insomnia patients (e.g., long sleep latency, substantial wake after sleep onset, and early morning awakenings). Moreover, like many insomnia patients, after night with those sleep patterns, there were following nights with longer sleep. Therefore, for some individuals, the night-to-night variability may not in itself be pathologic. It is possible that individuals with insomnia and an 8-h bedrest episode have similar patterns (Bianchi, 2017) that may contribute to complaints of short sleep duration.

### Ecological Significance

The question arises as to whether any relatively stable on average value in this sleep satiation protocol reflects sleep need for an individual person. If so, do all physiological processes (e.g., metabolism, immune function, mood regulation, subjective alertness) require the same amount of TST or NREM sleep? Or, does the TST or NREM sleep value reflect a ceiling of what the physiology allows or a compromise among multiple influences? Additional experiments are required to address these questions, including the definition and physiology underlying sleep need. One possibility is that sleep need is reflected by TST and sleep pressure is reflected by SWS. However, the fact that relative SWS is preserved in chronic sleep restriction studies (Belenky et al., 2003; Van Dongen et al., 2003; McHill et al., 2018) as neurobehavioral performance and glucose metabolism deteriorate, suggests that TST may be a better marker of sleep need for these physiological functions than SWS. Note, however, both TST and SWS require the individual to fall asleep before they can be expressed; if there are problems falling asleep [e.g., due to age-related changes in neuron number (Lu et al., 2000; Lim et al., 2014)], then these metrics may not accurately reflect sleep need or pressure.

In summary, data from two studies with extended sleep opportunities reveal consistent evidence of self-selected chronic sleep restriction in individuals living at home, and a multi-day time course of recovery from this chronic insufficient sleep. These individuals may not have recognized that their self-selected sleep patterns were not allowing for sufficient sleep. Once steady state-values were reached, there were large nightly variations in each metric. The steady-state value of TST was more than 8–1/2 h; no individual consistently slept more than 10 h. Therefore, there was no evidence that healthy people can sustain over multiple days, sleeping throughout long (e.g., > 10 h) bedrest episodes. Consistent sleep durations more than 10 h may have a pathophysiological basis (e.g., sleep disorder or other disease) that should be diagnosed and treated. The long sleep episodes that some people have on a free day and some people associate with over-sleeping are probably a transient self-limiting phenomenon, suggesting that people can not consistent over-sleep in the same way that they can consistently over-eat. Additional research is required to document inter-individual and intra-individual variability and how many nights are required for recovery of other physiologic and behavioral processes (e.g., mood, metabolism, learning) from insufficient sleep.

## Data Availability Statement

The original contributions presented in the study are included in the article/Supplementary Material, further inquiries can be directed to the corresponding author/s.

## Ethics Statement

The studies involving human participants were reviewed and approved by NIH Intramural Research Program IRB (Study 1) or Partner’s Healthcare IRB (Study 2). The patients/participants provided their written informed consent to participate in this study.

## Author Contributions

EK, GB, and TW conducted the experiments and were responsible for the primary analyses. CC and TW obtained the funding. EK conducted the secondary analyses. All authors contributed to writing and editing of the manuscript.

## Conflict of Interest

Financial Disclosure

EK (2019-present) has consulted for Pfizer Pharmaceuticals, the National Sleep Foundation, Sanofi-Genzyme, and Circadian Therapeutics; received travel support from Society for Reproductive Investigation, the Sleep Research Society, the National Sleep Foundation; The World Conference of Chronobiology, and the Gordon Research Conferences. She reviewed payment from the Puerto Rico Trust for a grant review, and received a grant from the Australia Harvard Club. GB (2019-present) has no outside funding to disclose. CC reports grants to BWH from FAA, NHLBI, NIA, NIOSH, NASA, and DOD; is/was a paid consultant to AARP, American Academy of Dental Sleep Medicine, Eisenhower Medical Center, Emory University, Inselspital Bern, Institute of Digital Media and Child Development, Klarman Family Foundation, M. Davis and Co., Physician’s Seal, Sleep Research Society Foundation, State of Washington Board of Pilotage Commissioners, Tencent Holdings Ltd., Teva Pharma Australia, UC San Diego, University of Washington, and Vanda Pharmaceuticals Inc., in which Czeisler also holds an equity interest; received travel support from Annenberg Center for Health Sciences at Eisenhower, Aspen Brain Institute, Bloomage International Investment Group, Inc., UK Biotechnology and Biological Sciences Research Council, Bouley Botanical, Stanley Ho Medical Development Foundation, European Biological Rhythms Society, German National Academy of Sciences (Leopoldina), Illuminating Engineering Society, National Safety Council, National Sleep Foundation, Society for Research on Biological Rhythms, Sleep Research Society Foundation, Stanford Medical School Alumni Association, Tencent Holdings Ltd., University of Zurich, and Vanda Pharmaceuticals Inc., Ludwig-Maximilians-Universität München, National Highway Transportation Safety Administration, Office of Naval Research, Salk Institute for Biological Studies/Fondation Ipsen; receives research/education support through BWH from Cephalon, Mary Ann & Stanley Snider *via* Combined Jewish Philanthropies, Harmony Biosciences LLC, Jazz Pharmaceuticals PLC Inc., Johnson & Johnson, NeuroCare, Inc., Philips Respironics Inc.,/Philips Homecare Solutions, Regeneron Pharmaceuticals, Regional Home Care, Teva Pharmaceuticals Industries Ltd., Sanofi SA, Optum, ResMed, San Francisco Bar Pilots, Sanofi, Schneider, Simmons, Sysco, Philips, Vanda Pharmaceuticals; is/was an expert witness in legal cases, including those involving Advanced Power Technologies, Aegis Chemical Solutions LLC, Amtrak; Casper Sleep Inc., C&J Energy Services, Catapult Energy Services Group, LLC, Covenant Testing Technologies, LLC, Dallas Police Association, Enterprise Rent-A-Car, Espinal Trucking/Eagle Transport Group LLC/Steel Warehouse Inc., FedEx, Greyhound Lines Inc.,/Motor Coach Industries/FirstGroup America, Pomerado Hospital/Palomar Health District, PAR Electrical Contractors Inc., Product & Logistics Services LLC/Schlumberger Technology Corp.,/Gelco Fleet Trust, Puckett Emergency Medical Services LLC, South Carolina Central Railroad Company LLC, Union Pacific Railroad, United Parcel Service/UPS Ground Freight Inc., and Vanda Pharmaceuticals; serves as the incumbent of an endowed professorship provided to Harvard University by Cephalon, Inc.; and receives royalties from McGraw Hill, and Philips Respironics for the Actiwatch-2 and Actiwatch Spectrum devices. Czeisler’s interests were reviewed and are managed by the Brigham and Women’s Hospital and Mass General Brigham in accordance with their conflict of interest policies. TW (2019-present) has no outside funding to disclose.

Non-Financial Disclosure

EK: partner owns Chronsulting. GB, CC, and TW: no non-financial disclosures.

## Publisher’s Note

All claims expressed in this article are solely those of the authors and do not necessarily represent those of their affiliated organizations, or those of the publisher, the editors and the reviewers. Any product that may be evaluated in this article, or claim that may be made by its manufacturer, is not guaranteed or endorsed by the publisher.
