# Supplementary material for: Can People Sleep Too Much? Effects of Extended Sleep Opportunity on Sleep Duration and Timing
Source: Front Physiol. 2021 Dec 22;12:792942. doi: 10.3389/fphys.2021.792942 (PMC8727775; doi:10.3389/fphys.2021.792942)

Can people sleep too much?
effects of extended sleep opportunity
on sleep duration and timing

Elizabeth B. Klerman^1,2*^, Giuseppe Barbato^3^, Charles A Czeisler^1^, Thomas Wehr^4^

^1^ Division of Sleep and Circadian Disorders, Brigham and Women’s Hospital; ^2^Present address: Department of Neurology, Massachusetts General Hospital; and Harvard Medical School, Boston MA

^3^Dept. Psychology, University degli Studi della Campania Luigi Vanvitelli, Caserta, Campania, Italy

^4^Intramural Research Program, NIMH, Bethesda MD

Supplementary Material

# Supplementary Data

N/A

# Supplementary Figures and Tables

## Figure Legends

**Supplementary Figure 1:** Raster plots of Study 1 (top) and example raster plot of Study 2 (bottom). For Study 2, the sleep timing was adjusted for each individual. In a raster plot, time of day is across the x-axis and days are on the y-axis. Dark bars indicate the time of a sleep episode; dashed bars indicate times of Multiple Sleep Latency Testing.

**Supplementary Figure 2:** As in Figure 1(A) for variables NREM Sleep Stages 1 and 2, SWS, Wake in PersistSlp, Final Wake Duration, and Sleep Episode Duration (= start of PersistSlp,to Final Wake Duration).

**Supplementary Figure 3:** As in Figure 3 except all 28 nights of Study 1 are plotted for TST, NREM sleep, SWS, REM Sleep and Wake.

**Supplementary Figure 4:** Color heat maps of 60-minute bins of TST for each participant for Study 1. 60-minute bins in which participants were awake the entire time are plotted as 0 minutes of TST. Participant numbers are the same as in Figures 4 and 5. Summaries for the last 14 days are plotted below each panel.

**Supplementary Figure 5:**  Individual daily variations in the difference between consecutive night’s metric (e.g., TST, Latency to PersistSlp,, NREM Sleep, REM Sleep) for the last 14 nights in Study 1. Consecutive values are joined by a line. Participant numbers are the same as in Figures 4 and 5.

## Supplementary Figures

**Supplementary Figure 1.**

**Supplementary Figure 2.**


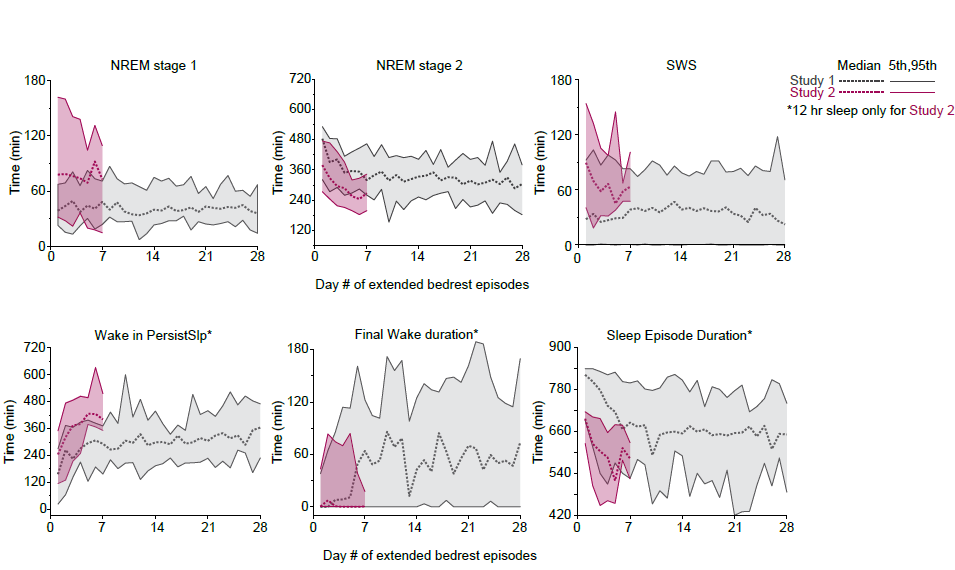


**Supplementary Figure 3.**


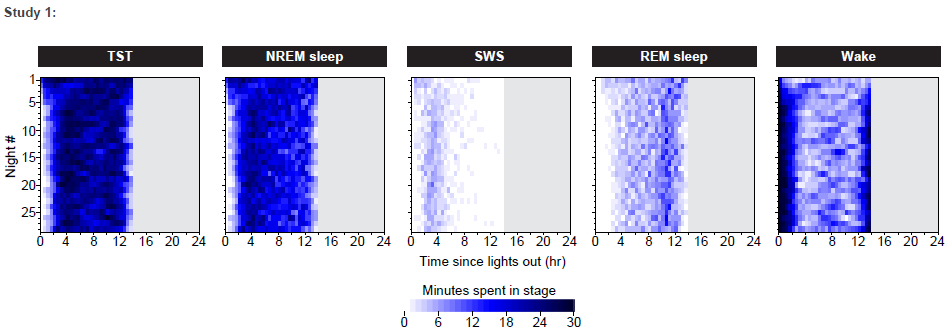


**Supplementary Figure 4.**


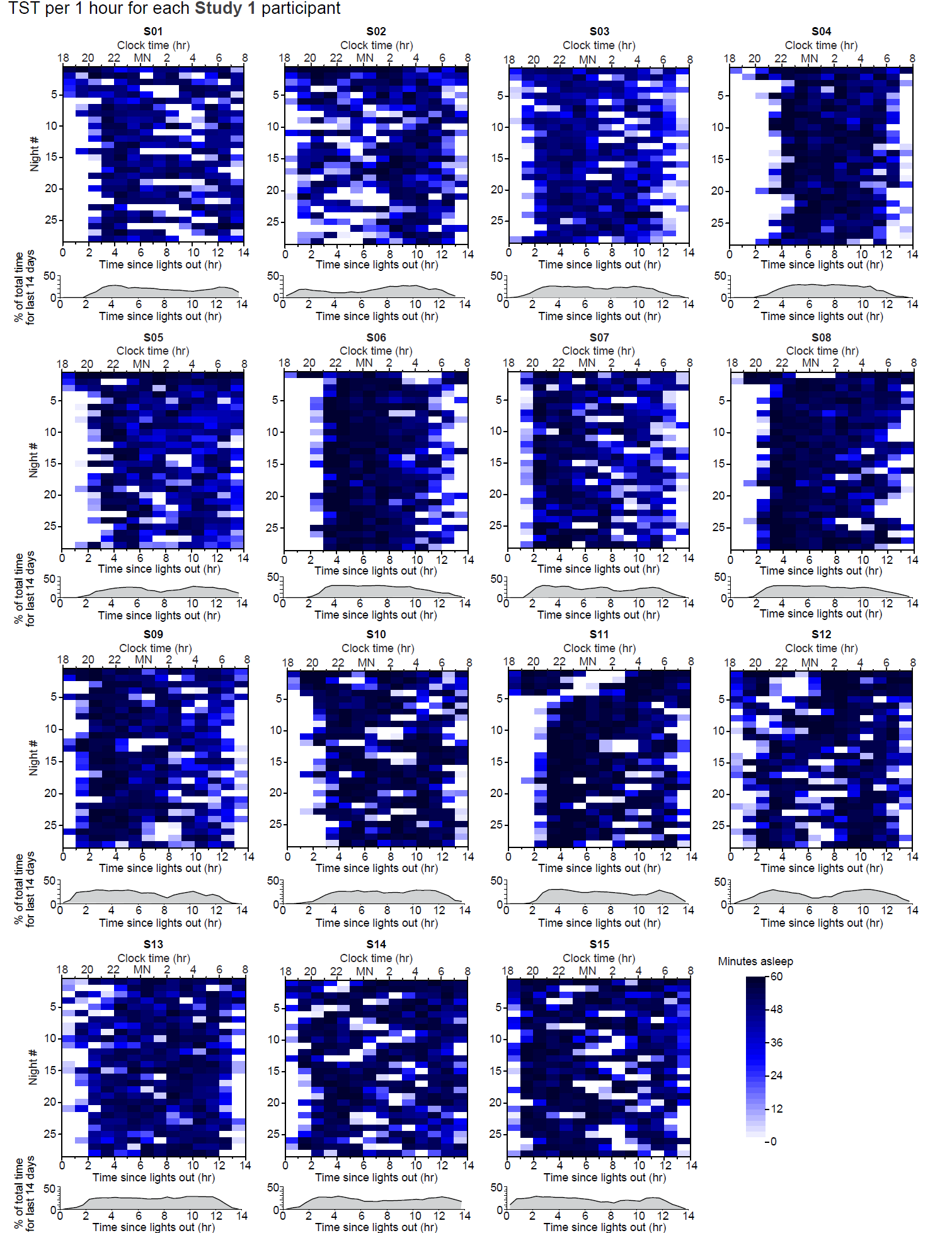


**Supplementary Figure 5.**

Study 1:


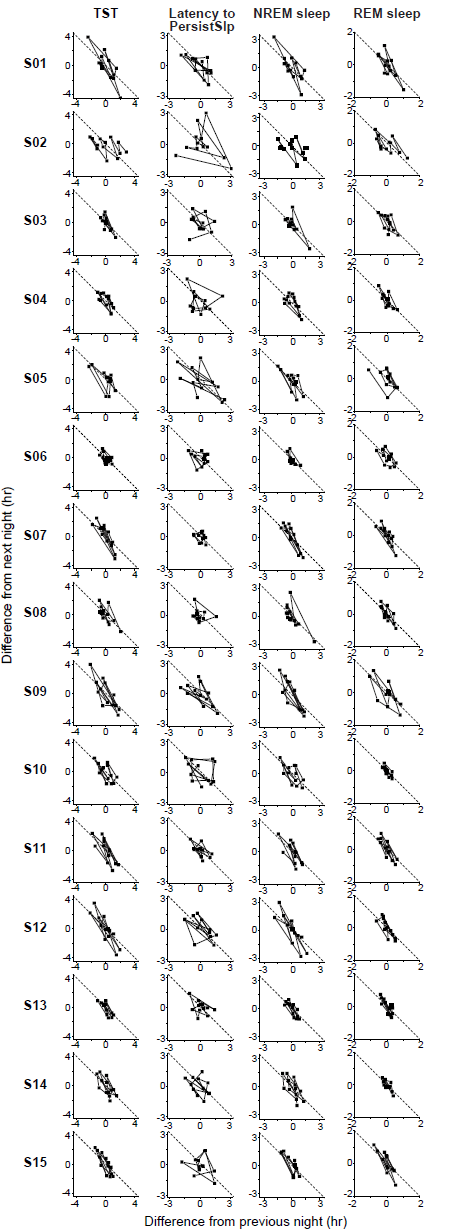

Supplement: Supplementary file 1 [file Data_Sheet_1.docx]
